# Familial Mediterranean fever with sigmoid colon stricture

**DOI:** 10.1007/s12328-025-02095-1

**Published:** 2025-01-10

**Authors:** Yuki Yamamoto, Akira Madarame, Masakatsu Fukuzawa, Tadashi Ichimiya, Yoshiya Yamauchi, Sakiko Naito, Takashi Morise, Yasuyuki Kagawa, Takahiro Muramastu, Takao Itoi

**Affiliations:** Department of Gastroenterology and Hepatology, Tokyo Medical University Hospital, 6-7-1, Nishi-shinjuku, Shinjuku-ku, Tokyo 160-0023 Japan

**Keywords:** Familial Mediterranean fever, Intestinal stricture, Crohn’s disease, Colchicine, Inflammatory bowel disease

## Abstract

We describe a case of familial Mediterranean fever (FMF) with sigmoid colon stricture. The patient, a woman in her 30 s, had a 12-year history of ileocolitis-type Crohn’s disease. The colonoscope could not pass because of the sigmoid colon stricture, and the patient was referred to our hospital with complaints of abdominal pain and fever. At 2-month postreferral, the patient presented with severe abdominal pain and fever. Computed tomography and intestinal ultrasonography revealed no bowel obstruction, whereas wall thickening was observed in the sigmoid colon and small bowel. Our medical interview revealed a cyclical nature to the symptoms. We diagnosed FMF and initiated colchicine. Subsequently, for more than 2 years, the patient remained asymptomatic, and the sigmoid colon stricture improved. FMF should be considered in patients with inflammatory bowel disease with periodic abdominal pain and fever.

## Introduction

Familial Mediterranean fever (FMF) is an autoinflammatory disease characterized by *MEFV* mutations, leading to periodic fever with serositis. The *MEFV* gene, which encodes pyrin, is a protein involved in regulating inflammation. FMF is highly prevalent among ethnic groups originating from the Mediterranean region, including Jews from the Middle East and North Africa, Armenians, and Turks [1]. In recent years, reports of FMF in Japan have increased, emphasizing the expanding recognition and diagnosis of this disease beyond its traditional geographic boundaries [2].

The inappropriate activation of the innate immune system, leading to the excessive production of proinflammatory cytokines, particularly interleukin-1β (IL-1β), constitutes the pathogenesis of FMF. This cytokine storm causes the characteristic clinical symptoms of fever and serositis. The common manifestations of serositis include pericarditis, pleuritis, and peritonitis, which can cause episodes of severe chest and abdominal pain with fever and significantly affect the patient’s quality of life [3].

Interestingly, several studies have suggested that FMF is complicated with inflammatory bowel disease (IBD) [4, 5], indicating a higher IBD prevalence in patients with FMF [5]. Some patients with *MEFV* mutations who respond to colchicine have endoscopic findings resembling IBD [6]. These coincidences and similarities complicate FMF diagnosis and management, thereby leading to patients being treated for IBD without recognizing the underlying FMF.

We here present a case of Crohn’s disease (CD) initially diagnosed with sigmoid colon stricture, which was subsequently diagnosed of FMF owing to the presence of periodic fever and abdominal pain. As there have been no previous reports of FMF presenting with sigmoid colon strictures, this case is particularly notable. Our findings suggest that colchicine, a mainstay treatment for FMF, was effective in controlling the periodic inflammatory episodes while managing the colonic stricture. This case emphasizes the significance of including FMF in the differential diagnosis of patients presenting with recurrent fevers and gastrointestinal manifestations.

## Case report

**Fig. 1:**
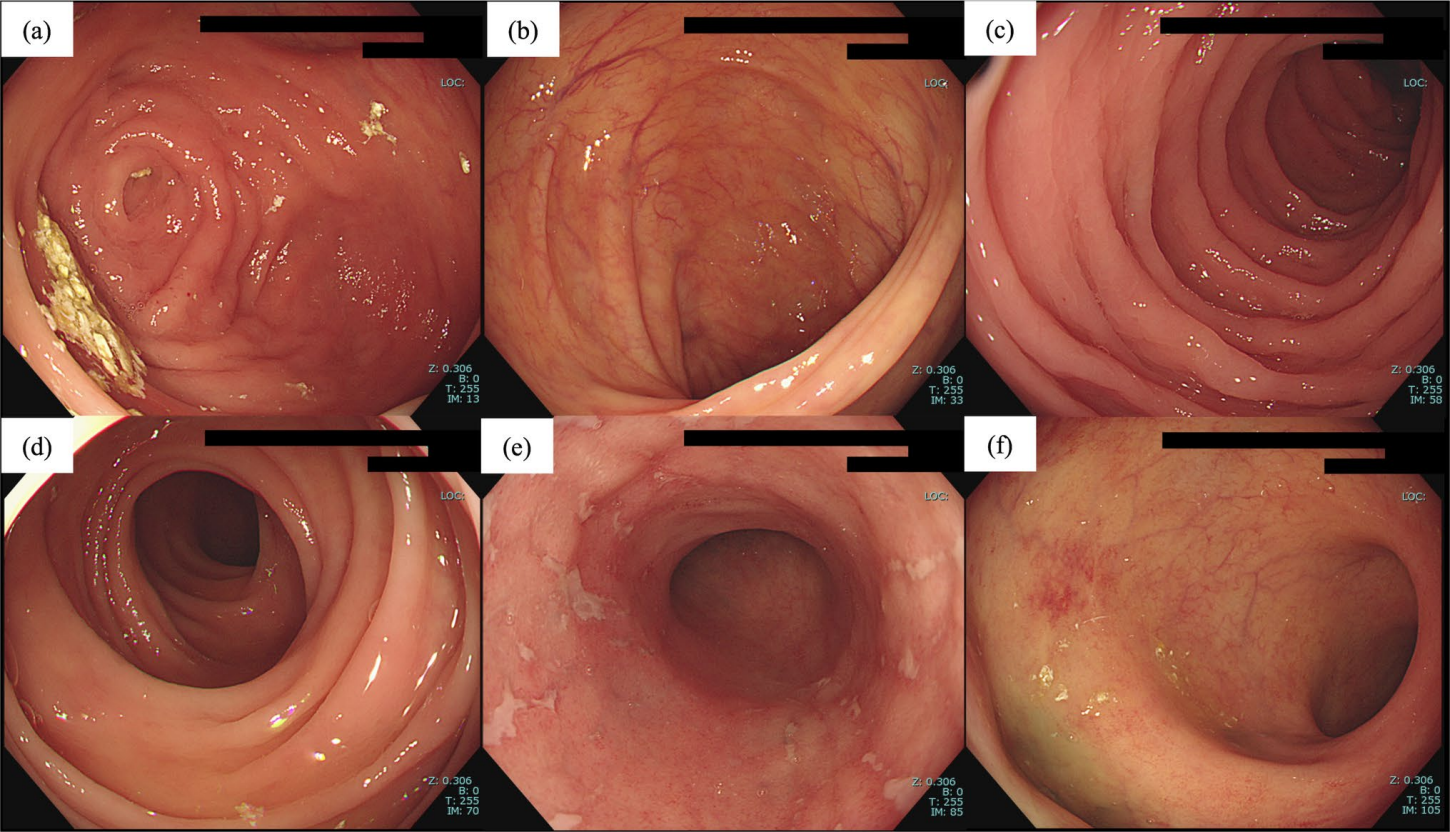
Image of the colonoscopy performed 3 years before her visit to the hospital while on 5-aminosalicylic acid and azathioprine treatment. **a** Cecum. **b** Ascending colon. **c** Transverse colon. **d** Descending colon. From the cecum to the descending colon, the findings were normal colon mucosa. **e** Sigmoid colon. Scattered longitudinal erosions were observed. **f** Rectum. Erythema and aphthae were detected

**Fig. 2:**
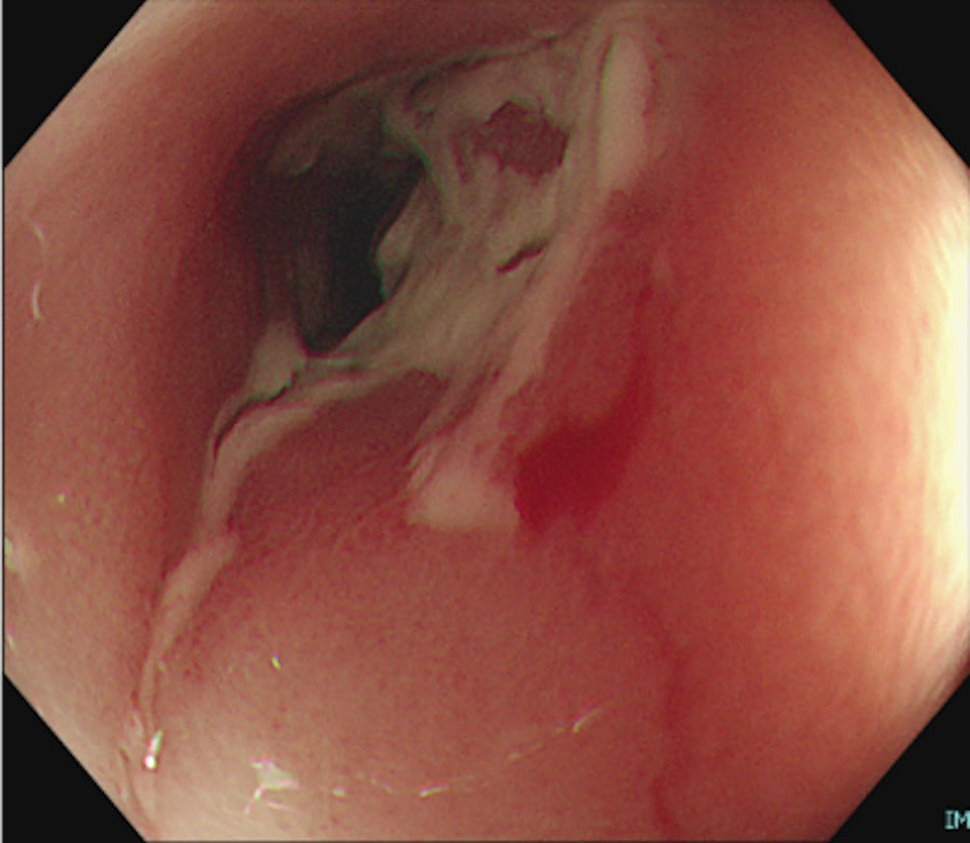
Sigmoid colon 2 months before the hospital visit. Stricture with ulceration prevented the passage of the colonoscope

The patient was a woman in her 30 s who was diagnosed with ileocolitis-type CD 12 years ago and was administered azathioprine 100 mg and 5-aminosalicylic acid 3 g daily. Her medical history was notable for depression and atopic dermatitis, without familial predisposition to IBD. Fever and abdominal pain were recurrent, which were ameliorated with short-term steroid treatment. Colonoscopy performed 3 years before her visit to our hospital revealed longitudinal erosions in the sigmoid colon, erythema, and aphthae in the rectum. The mucosa from the appendix to the descending colon was normal (Fig. 1). Two months before the patient visited our hospital, a sigmoid colon stricture with a longitudinal ulcer was detected, obstructing the passage of the colonoscope (Fig. 2). However, esophagogastroduodenoscopy showed normal findings. The patient presented to our hospital with the chief complaint of abdominal pain and fever. Two months after the visit, the patient developed severe abdominal pain and fever, requiring urgent hospitalization.

**Fig. 3:**
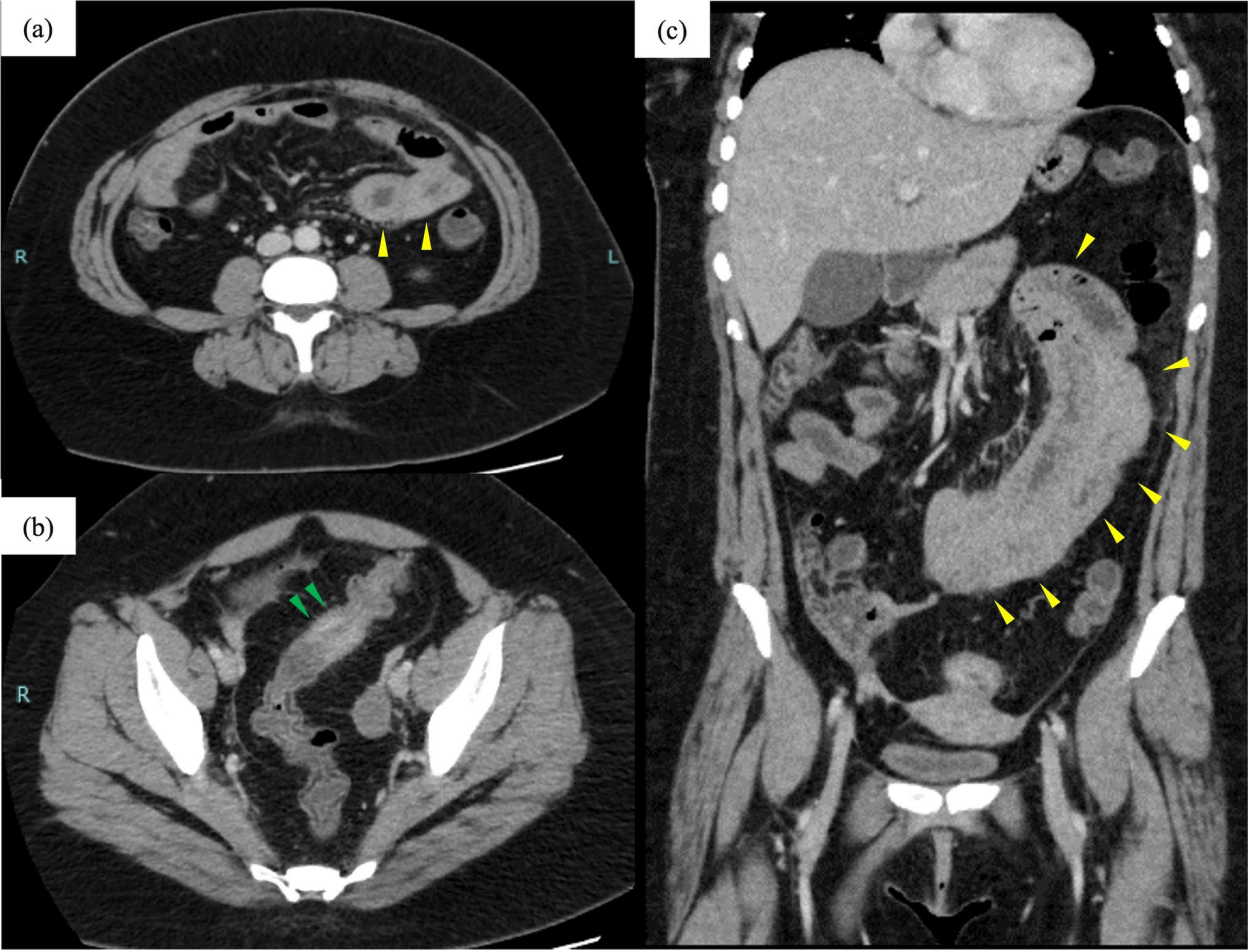
Computed tomography image at the time of exacerbation. Intestinal wall thickening with contrast effect was observed in the sigmoid colon and small intestine. The jejunum and sigmoid colon are indicated by the yellow and green arrowheads, respectively. **a** Horizontal section at the level of the jejunum. **b** Horizontal section at the level of the Sigmoid colon. **c** Coronary section

**Fig. 4:**
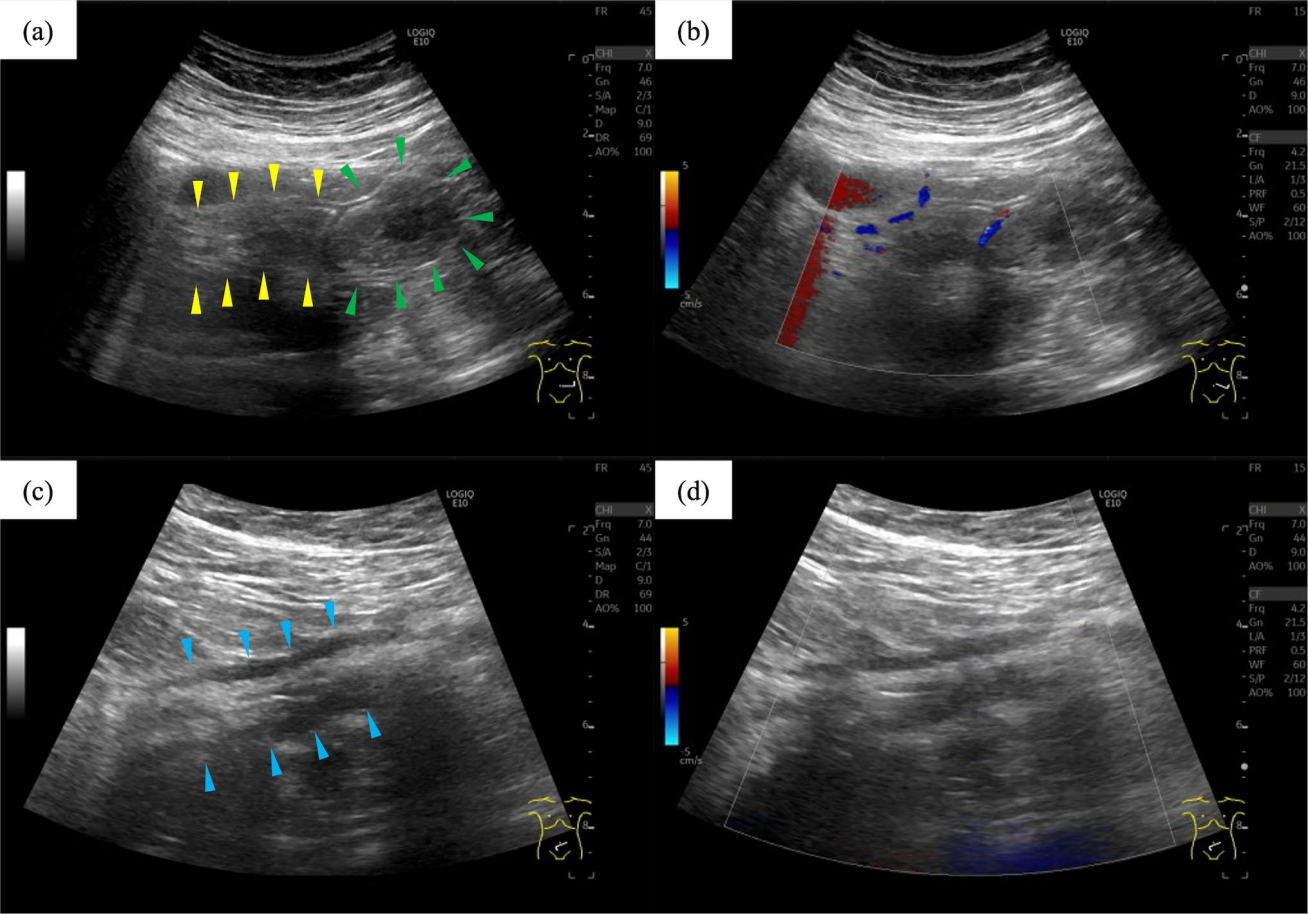
Intestinal ultrasonography image at the time of exacerbation. **a** The jejunum in the long axis image and the short axis image were indicated by the yellow and green arrowheads, respectively. Intestinal wall thickening was noted. **b** Color Doppler showed that blood flow was increasing on the serosal side of the jejunum (velocity range: 5 cm/second). **c** The sigmoid colon in the long axis image is indicated by the blue arrow. Intestinal wall thickening was observed. **d** Color Doppler showed no blood flow signals in the sigmoid colon (velocity range: 5 cm/second)

On admission, physical examination showed upper abdominal guarding and rebound tenderness. Blood test results revealed a white blood cell (WBC) count of 10,700/μL and a C-reactive protein (CRP) level of 13.1 mg/dL. The serum amyloid A (SAA) level was 1340 μg/mL (Table 1). Stool bacterial culture yielded no growth of pathogenic organisms. Computed tomography (CT) revealed no bowel obstruction, whereas wall thickening was noted in the sigmoid colon and small bowel (Fig. 3). Intestinal ultrasonography revealed wall thickening in the ileum and sigmoid colon. The color Doppler mode exhibited increased blood flow on the serosal side of the thickened small intestinal wall, whereas the thickened sigmoid colon wall showed no increased blood flow (Fig. 4).

**Table 1:** Laboratory findings at the time of exacerbation

WBC	10,700 /µl	AST	17 IU/l	K	3.9 mEq/l
Seg	90.1%	ALT	8 IU/l	Cl	99 mEq/l
Eosino	0%	LDH	260 IU/l	BS	121 mg/dl
Baso	0.2%	ALP	73 IU/l	CK	61 U/l
Lymph	7.1%	γ-GTP	13 IU/l	CRP	13.1 mg/dl
Mono	2.6%	T-Bil	0.85 g/dl	SAA	1340 μg/ml
RBC	434 × 10^4^/μl	Alb	4.0 mg/dl		
Hb	13.2 g/dl	BUN	6.1 mg/dl		
Ht	38.9%	Cr	0.48 mg/dl		
Plt	308 × 10^3^/μl	Na	134 mEq/l		

*WBC* white bold cell, *Seg* segmented neutrophils, *Eosino* eosinophils, *Baso* basophil, *Lymph* lymphocytes, *Mono* monocytes, *RBC* red blood cell, *Hb* hemoglobin, *Ht* hematocrit, *Plt* platelets, *AST* aspartate aminotransferase, *ALT* alanine aminotransferase, *LDH* lactate dehydrogenase, *ALP* alkaline phosphatase, *γ-GTP* γ-glutamyl transferase, *T-bil* total bilirubin, *Alb* albumin, *BUN* blood urea nitrogen, *Cr* creatinine, *Na* sodium, *K* potassium, *Cl* chloride, *BS* blood sugar, *CK* creatin kinase, *CRP* C-reactive protein, *SAA* serum amyloid A

**Fig. 5:**
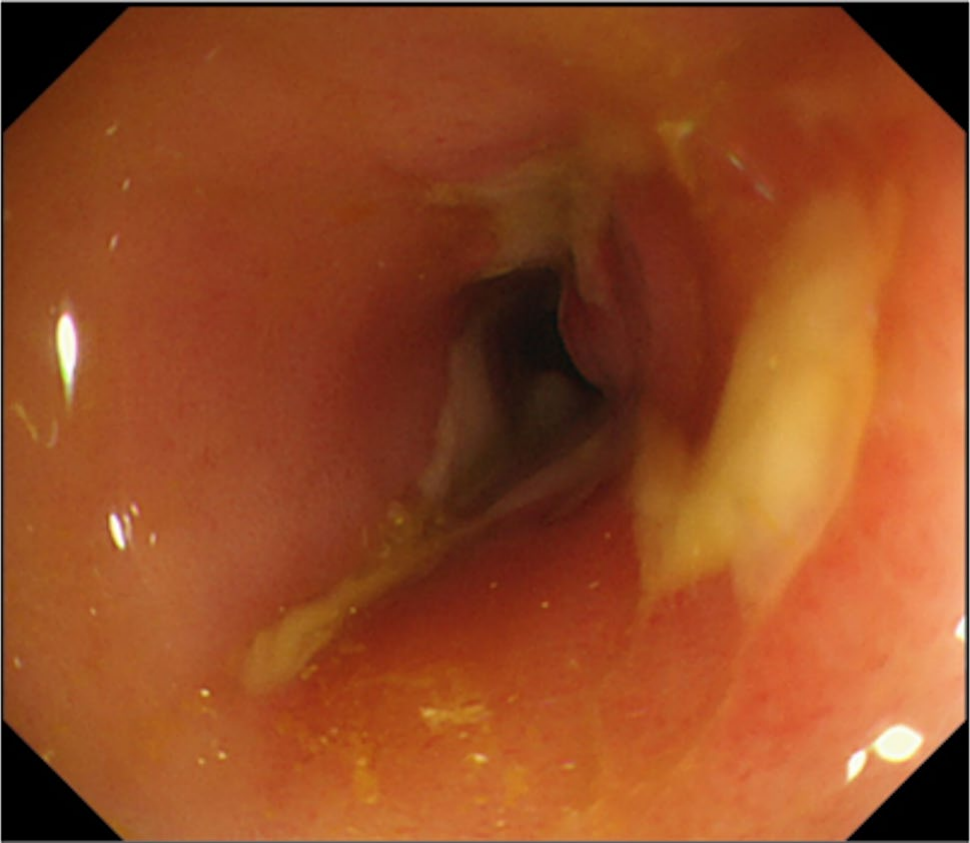
Sigmoid colon on hospital day 8. Compared with Fig. 2, no significant change was observed

**Fig. 6:**
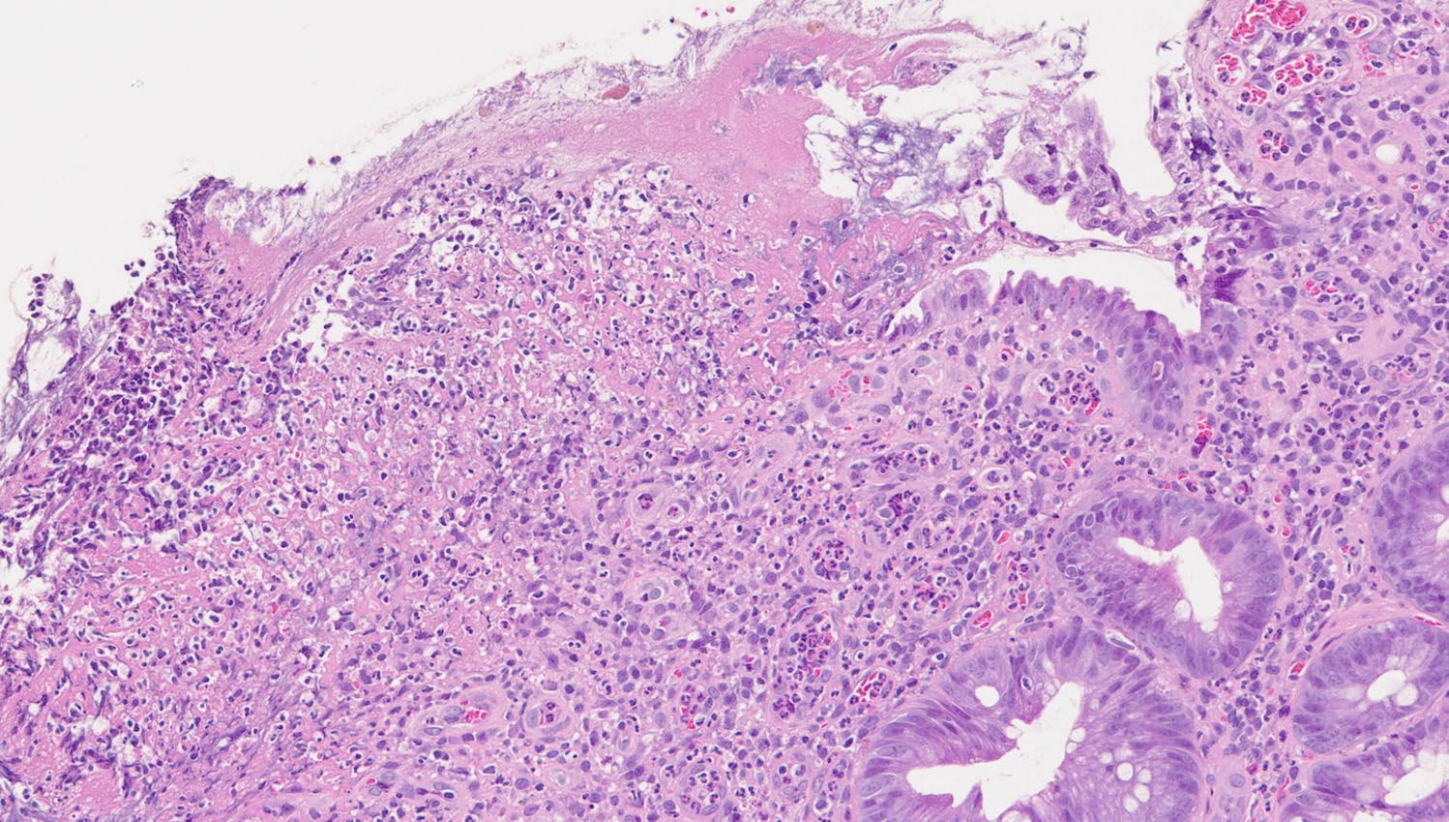
Histologic analysis of biopsy specimens obtained from the ulcer of the sigmoid colonic mucosa on hospital day 8. A diffuse inflammatory cell infiltrate with neutrophils, lymphocytes, and plasma cells is observed, with no presence of granulomas (Hematoxylin & eosin, X200)

Following admission, treatment comprised fasting and intravenous fluid infusion. On hospital day 4, the abdominal pain and fever disappeared. On hospital day 8, sigmoidoscopy revealed no worsening of the ulcer in the sigmoid colon compared with the previous findings (Fig. 5). Histologic findings of biopsy specimens obtained from the sigmoid colon ulcer showed neutrophil, lymphocyte, and plasma cell infiltration, and these findings were consistent with those of CD (Fig. 6). According to our medical interview, periodic fever and abdominal pain lasting for several days monthly were recurrent since being diagnosed with CD. FMF was suspected, and 1 mg of oral colchicine daily was administered as diagnostic treatment. On hospital day 20, the patient was discharged without any worsening of symptoms.

**Fig. 7:**
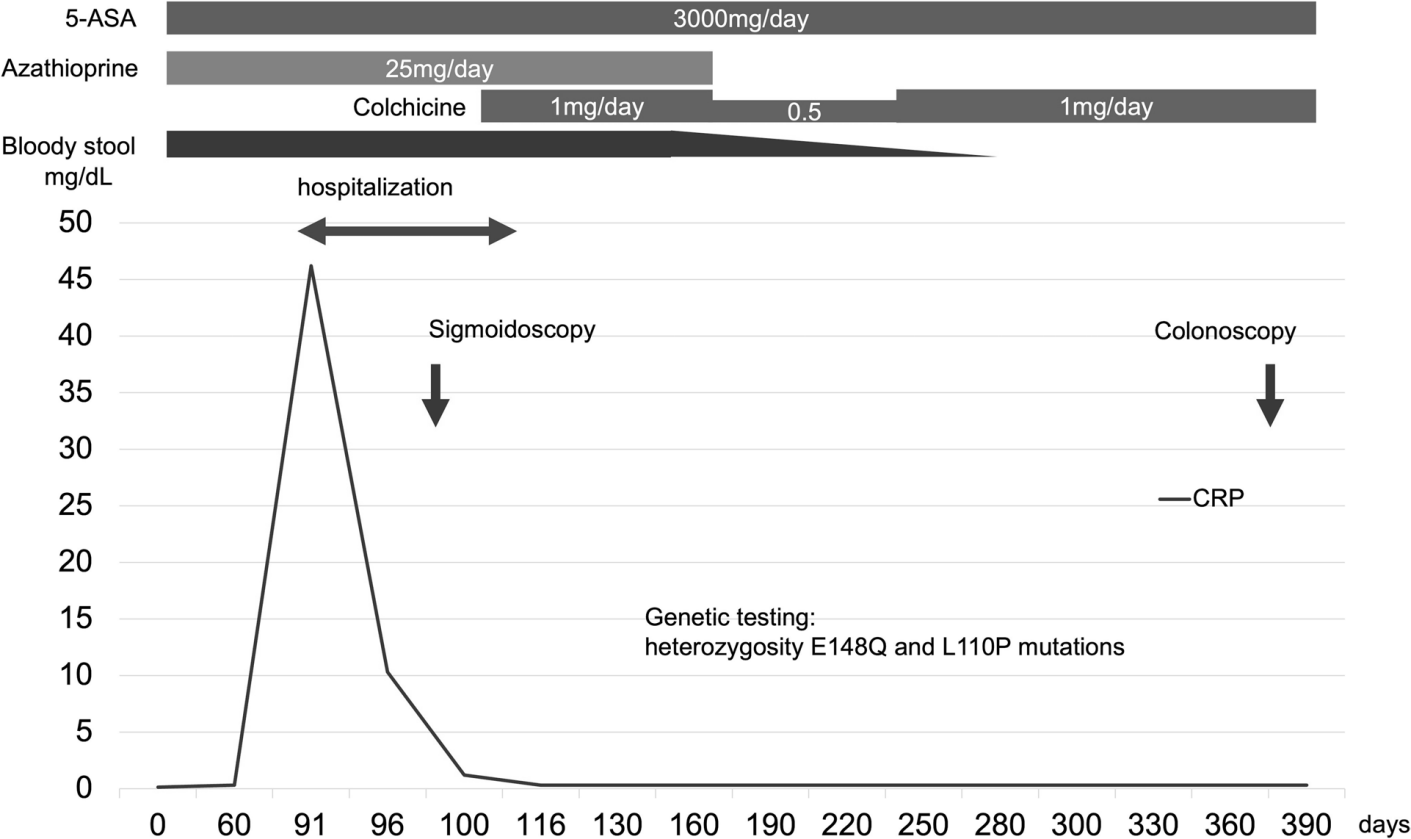
Clinical course

**Fig. 8:**
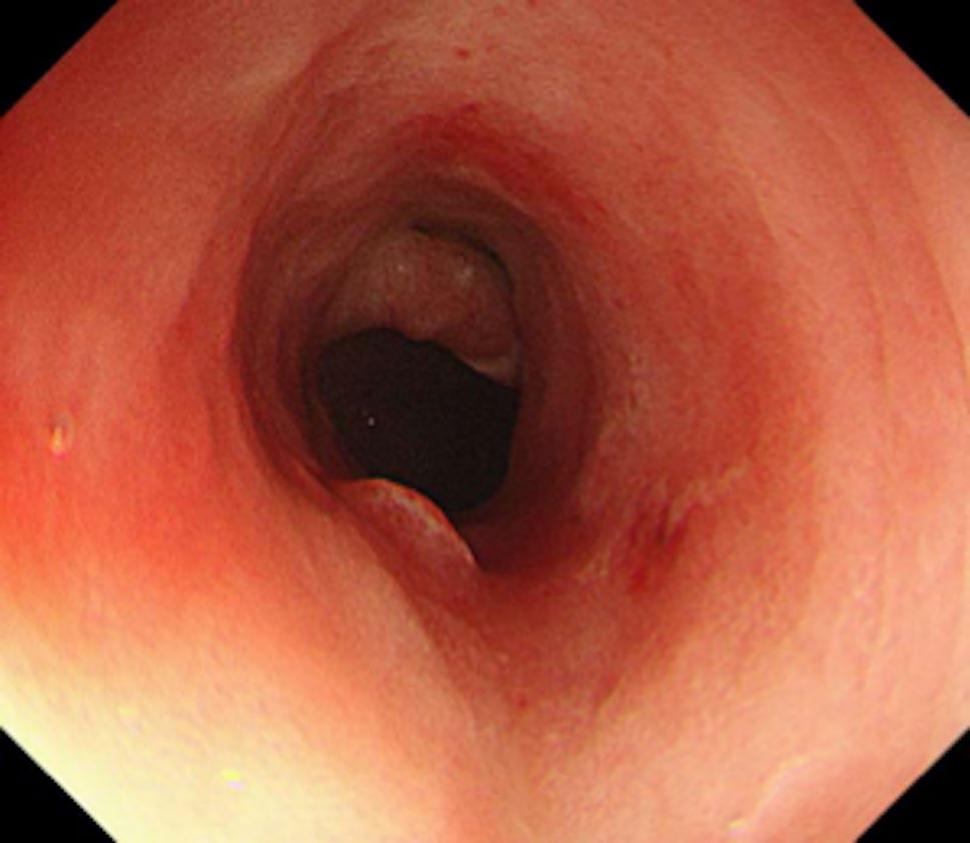
Sigmoid colon 10 months after colchicine initiation. The ulcer has disappeared, and the colonoscope can be passed through

Subsequently, genetic testing identified heterozygosity E148Q and L110P mutations on the second exon of the *MEFV* gene. Myelosuppression developed following colchicine initiation; therefore, azathioprine was discontinued, and the colchicine dose was reduced to half. 5-ASA was continued because 5-ASA-induced myelosuppression was infrequent and discontinuation of 5-ASA in the case of concomitant CD could worsen the stricture. The WBC counts improved, and the colchicine dose was returned to 1 mg/day. For 2 years following colchicine initiation, the patient had no periodic symptoms (Fig. 7). At 10 months following colchicine initiation, colonoscopy revealed that the ulcer in the sigmoid colon had disappeared, and the colonoscope could pass through the sigmoid colon (Fig. 8).

## Discussion

We presented a case with FMF and sigmoid colon stricture. The improvement of the sigmoid colon stricture following colchicine administration was remarkable. This case may yield valuable insights for identifying patients with FMF among those with CD.

FMF is an inherited disease diagnosed according to the Tel-Hashomer criteria, and it is believed that there are more than 100,000 patients with FMF worldwide [7]. According to this criterion, FMF is divided into two types, “typical” and “atypical,” on the basis of clinical findings. Fever, abdominal pain, chest pain, and frequently joint pain and rash due to peritonitis are the main symptoms of FMF [1], and the differential diagnosis between FMF and CD is very challenging owing to the highly similar clinical presentations.

Here, FMF was suspected owing to repeated episodes of fever and abdominal pain lasting for several days monthly; however, the patient was initially diagnosed with CD with sigmoid colon stricture. During the episodes, CRP and SAA levels markedly increased, and CT and intestinal ultrasonography revealed extensive wall thickening of the small intestine and sigmoid colon. Furthermore, muscular defense and rebound pain due to peritonitis were confirmed, and the patient was diagnosed as a typical case of FMF. Colchicine was highly effective, and after visiting our hospital, the patient’s fever and abdominal pain disappeared for more than 2 years. Moreover, the bloody stools improved, and the sigmoid colon ulcer that hindered the passage of the colonoscope disappeared, thereby enabling the colonoscope to pass through.

In FMF, the function of pyrin, which activates inflammasomes and promotes caspase-1, IL-1β, and IL-18 production, is impaired owing to *MEFV* gene mutations, thereby resulting in inflammation. Pyrin plays a role in the permeability of the gastrointestinal mucosa, which is believed to contribute to gastrointestinal lesion formation [8]. The *MEFV* gene comprises 10 exons, and mutations in exon 10 of the *MEFV* gene, including M694V, V726A, and M694I, are common in patients with FMF in the Mediterranean region [9]. Conversely, mutations, including M694I, E148Q, L110P/E148Q, and P369S/R408Q, are more common in Japan [2]. Dimitri et al. argued that the E148Q mutation was a benign polymorphism because the frequency of the mutation was the same in patients with FMF and asymptomatic relatives [10]. Separately, it was reported that patients with the E148Q mutation showed the typical FMF phenotype [2]. Heterozygosity for the E148Q and L110P mutations was observed in the present case who showed typical FMF symptoms.

Several reports on endoscopic findings in FMF have been noted; however, no specific and consistent mucosal findings have been identified. Arasawa et al. reported pseudopolypoid-like lesions in the transverse colon [6], and Yokoyama et al. reported longitudinal ulcers in the terminal ileum and erythema, erosions, and ulcers in the ascending colon [11]. A study on the colonoscopy findings of eight cases of IBD unclassified (IBDU) with *MEFV* gene mutations revealed various mucosal lesions, rectal sparing, right-sided dominant colitis, pseudopolyposis, and granular protrusions [12]. Ezaki et al. reported a case of *MEFV* gene mutation-associated duodenojejunal pseudopolyposis [13]. Demir et al. reported 41 cases of FMF in which small-bowel lesions were diagnosed using capsule endoscopy and detected several erosions and ulcers in the jejunum [14]. Shibata et al. observed discontinuous loss of vascular markings, erosions, and friable mucosa, similar to ulcerative colitis, except in the rectum [15]. From these reports, the endoscopic findings of FMF may be analogous to those of IBD, and distinguishing between these diseases on the basis of endoscopic findings is difficult. This case showed a sigmoid colon stricture with longitudinal ulceration resembling CD. We considered performing small intestine examinations; however, even though the sigmoid colon stricture had improved, we considered it too narrow to perform a transanal balloon-assisted endoscopy. Moreover, the patient did not request a transoral balloon-assisted endoscopy or small-bowel capsule endoscopy as her symptoms had calmed down. Intestinal ultrasonography was performed during the critical phase, and although increased blood flow was observed on the serous side of the jejunum, no color Doppler signals were noted in the intestinal wall. No reports on the findings of intestinal ultrasonography in FMF are available; however, as increased blood flow in all layers was observed in the active phase of CD [16], it was suggested that increased blood flow on the serosal side can be useful in diagnosing FMF.

Regarding histologic findings, Agin et al. reported that 31% of patients with FMF who underwent colonoscopy had histologic findings suggestive of IBD, including goblet cell depletion, cryptitis, and crypt hyperplasia in the colon and terminal ileum [17]. In this case, noncaseating granulomatous cells were not detected; however, we confirmed the presence of cryptitis and diffuse inflammatory cell infiltration as observed in IBD. Therefore, as the histopathological differences between FMF and IBD have not been clearly elucidated, further research is warranted.

In this case, although FMF alone was the probable cause, the possibility that it was caused by a combination of FMF and CD cannot be excluded owing to prior endoscopic evidence of sigmoid colon narrowing. Twenty-five of 69 Armenian patients with IBD had FMF [18]. In a recent retrospective cohort study in Japan, MEFV mutations were detected in 238 of the 396 patients diagnosed with IBDU, with exon 2 mutations being the most common. Of the 134 cases, except for those with insufficient information on the clinical background and colchicine responsiveness, typical FMF and atypical FMF were 58 and 59 cases, respectively [19]. Gucenmez et al. reported that patients with FMF had significantly higher fecal calprotectin levels than those of healthy controls, suggesting that patients with FMF have asymptomatic enteritis [20]. In FMF, the suppression of caspase activity by pyrin is disrupted, thereby leading to an increased production of inflammatory cytokines, including IL-1β [8, 21]. Some reports have suggested that IL-1β induces IL-23, which is involved in refractory IBD [22, 23]. In addition, in IBD, inflammasomes, which secrete NLRP3, IL-1β, and IL-18, is involved in intestinal inflammation and fibrosis [24]. Colchicine has been demonstrated to inhibit the NLRP3 inflammasome and suppress caspase-1 activation in gout [25]. Therefore, colchicine also exhibits therapeutic potential in managing sigmoid colon ulcers and strictures.

Based on this case of a patient with sigmoid colon stricture who was successfully treated with colchicine after FMF was diagnosed following CD, the possibility of FMF should be considered when periodic exacerbations are observed.
